# Receivers Limit the Prevalence of Deception in Humans: Evidence from Diving Behaviour in Soccer Players

**DOI:** 10.1371/journal.pone.0026017

**Published:** 2011-10-05

**Authors:** Gwendolyn K. David, Catriona H. Condon, Candice L. Bywater, Daniel Ortiz-Barrientos, Robbie S. Wilson

**Affiliations:** School of Biological Sciences, The University of Queensland, St Lucia, Queensland, Australia; University of Osnabrueck, Germany

## Abstract

Deception remains a hotly debated topic in evolutionary and behavioural research. Our understanding of what impedes or facilitates the use and detection of deceptive signals in humans is still largely limited to studies of verbal deception under laboratory conditions. Recent theoretical models of non-human behaviour have suggested that the potential outcome for deceivers and the ability of receivers to discriminate signals can effectively maintain their honesty. In this paper, we empirically test these predictions in a real-world case of human deception, simulation in soccer. In support of theoretical predictions in signalling theory, we show that cost-free deceit by soccer players decreases as the potential outcome for the signaller becomes more costly. We further show that the ability of receivers (referees) to detect deceptive signals may limit the prevalence of deception by soccer players. Our study provides empirical support to recent theoretical models in signalling theory, and identifies conditions that may facilitate human deception and hinder its detection.

## Introduction

Deception is ubiquitous throughout human society and the animal kingdom, and remains a central theme in behavioural and evolutionary biology [1], [2], [3], [4], [5]. Deception occurs when an individual benefits from signalling false information to a receiver [6]. In humans, deceptive signals can be intentional and used to convey false information to manipulate a receiver. Since scientific observers share the same sensory system as their human subjects, identifying deception is easier than in nonhuman systems [7], [8]. However, our understanding of human deception is largely restricted to verbal signals and laboratory conditions due to ethical limitations in human research [9]. Identifying incentives for using deceit and improving the detection of deception in humans has received growing interest because of the clinical and legal applications [10], [11], [12], [13].

The cost of signals is the most widely accepted mechanism thought to maintain honesty in animal communication [14], [15], [16], [17]. Recent theoretical models have demonstrated that the potential cost of deception, not just signal production costs, can maintain honesty under conflict of interest [15], [18], [19], [20], [21], [22]. As long as the potential cost of deception is greater than the potential benefit, signallers will refrain from using deceptive signals [15]. There are several mechanisms whereby honesty could be maintained using the potential cost of deception, which largely rely on how receivers discriminate and respond to signals. Firstly, punishment of deceivers can deter the use of deception [5], [23]. Secondly, negative frequency-dependant selection strengthens correct discrimination of signals by receivers, as deceivers only benefit when rare in a population [24], [25]. Finally, close spatial proximity to signallers can improve a receiver's ability to detect and discriminate among signals [20], [21], [22], [26]. Under these mechanisms, how receivers respond to signals can greatly alter the potential outcome for deceivers and therefore the prevalence of deception. Despite the potential for identifying conditions that impede or facilitate the use and detection of human deception, animal signalling theory has not been applied to humans in real-world scenarios.

Professional sport is a real-world scenario that provides an opportunity to investigate human deception using animal signalling theory. In sport, players use deception to manipulate the behaviour of the opposition or referee to gain a competitive advantage [11], [27], [28]. In soccer, simulation behaviour (‘taking a dive’) is notorious and has substantial impacts on the quality of a match, its perceived fairness, and the game's worldwide marketability. A ‘dive’ (deceptive signal) is synonymous with animal mimicry, and occurs when a player (signaller) intentionally mimics the behaviour of an illegal tackle-induced fall (reliable cue) and the referee (receiver) responds as if it were a tackle-induced fall by rewarding the player with a free kick (signaller benefit). A tackle-induced fall, herein referred to as a tackle, is treated as a reliable cue rather than an honest signal, as signalling players can not choose to produce a tackle, it is forced upon them [5]. Dives are considered cost-free deceptive signals, as the cost and benefit of deception are solely dependent on how referees respond to the signal and not incurred from the production of the signal itself.

Here, we use behavioural deceit in soccer players, to empirically test predictions drawn from animal signalling theory [15], [18], [19], [20], [21], [22]. Specifically, we test whether the potential outcome for deceptive signallers and receiver ability to discriminate among signals, reduces the use of deception when interests conflict. In support of animal signalling theory [15], [18], [19], [20], [21], [22], we show that cost-free deceit by soccer players decreases as the potential outcome for the signaller is more costly or, less beneficial. Further, we find the ability of receivers to detect deception varies with spatial proximity and culture.

## Methods

This study was approved by the Human Experimentation Ethics Review Committee (HEERC) at the University of Queensland as the work conducted used pure observation of human behaviour that occurred in an open forum to the general public whereby observed subjects were not identified in the dataset. The HEERC waived the need for consent from observed subjects.

Ten televised matches from six high-profile professional soccer leagues were assessed using real-time, replay, slow-motion and multi-angle high definition footage (*n* = 60). A single observer categorised every fall as a: ‘dive’ (player falls intentionally mimicking the effects of a tackle whereby minimal or no contact was made by an opponent), ‘tackle’ (player falls because of the contact made by opponent) or ‘NA’ (player falls of their own accord, and no intentional mimicking of a tackle is used; i.e. loses balance, trips over, roles an ankle etc.). Non-referee observers using television replays have been previously shown to be capable of reliably distinguishing between dives and non-dives, as well as reliably assessing the intentions of falling players [28]. We quantified the repeatability of our fall categorisation by re-examining ten falls selected at random from every match. Overall repeatability of categorising falls was 99.8± 0.002%, with the repeatability for ‘dive’, ‘tackle’ and ‘NA’ fall categories being 100, 100 and 90%, respectively. ‘NA’ falls were only recorded in the first nine analysed matches to gain high categorisation repeatability, and then ignored subsequently due to irrelevance to the investigated outcomes. In all statistical analyses only dive and tackle falls were used.

For every categorised fall the following variables were noted: pitch zone, signaller team score, match time, league and referee response. Of the 2803 falls observed across sixty soccer matches, 169 (6%) were dives (deceptive signals) and 2633 (94%) were tackles (reliable cues). The mean frequency of dives and tackles per match were 3.0±0.2 and 44.0±1.1, respectively (*n* = 60). On average, referees rewarded 1.0±0.1 (33%) dives and 20.0±0.1 (45%) tackles per match. The occurrence of dives and tackles were not correlated (rho = 0.138, *n* = 60, *P* = 0.2917). Therefore, the choice by soccer players to use dives was not associated with the occurrence of tackles.

**Figure 1:**
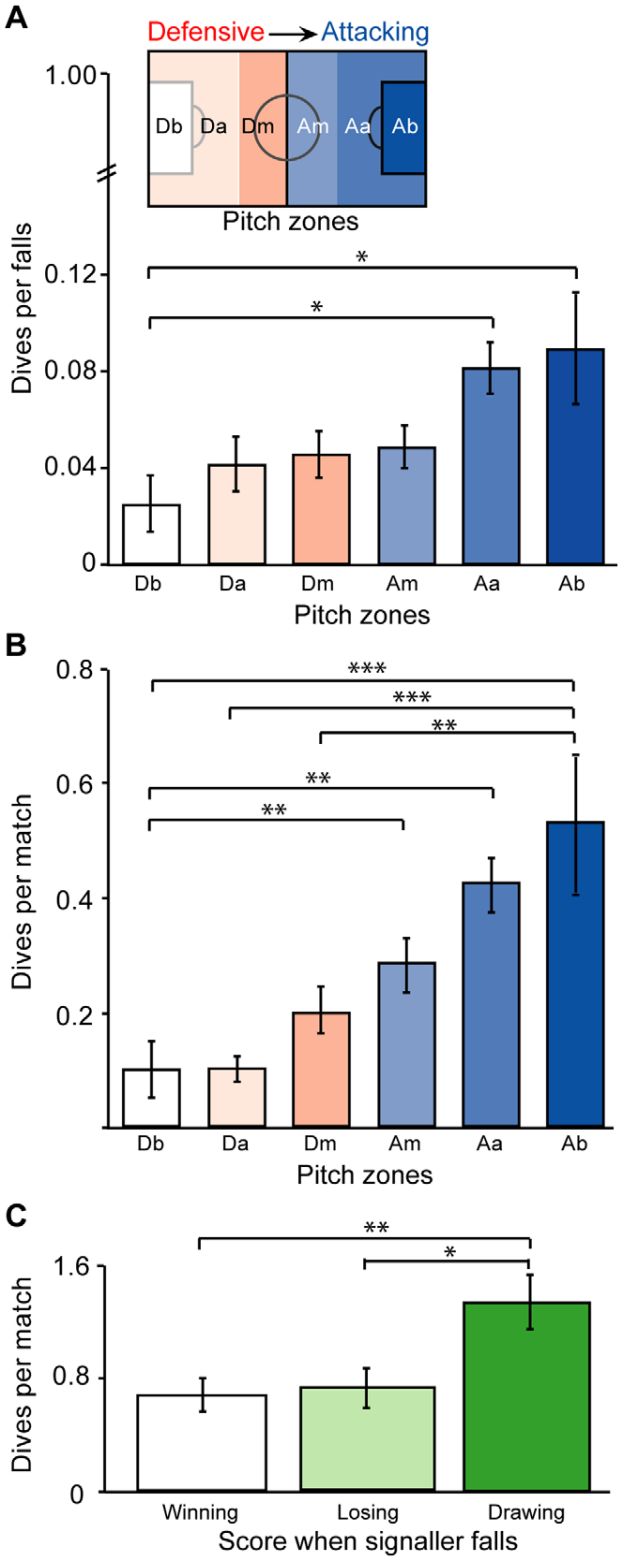
Deception across pitch zones and team scores. Dive use by soccer players is expressed as (A) the mean proportion of total falls per match that were dives; (B) the mean frequency of dives per match across pitch zones (corrected for spatial area) ordered by increasing distance from the defensive goal (see insert); and, (C) the mean frequency of dives per match that were signalled when the player was winning, losing or drawing. Standard error bars are presented (*n* = 60). Asterisks denote significant differences based on post-hoc Tukey-Kramer HSD (*P*<0.05; **P<0.01; ***P<0.001).

The soccer pitch was divided into six zones ordered by increasing proximity to the attacking goal (Fig. 1a, insert): Db (defensive box), Da (defensive area), Dm (defensive middle), Am (attacking middle), Aa (attacking area) and Ab (attacking box). Any fall that occurred on the penalty-box line was treated as the second furthest zone from the respective goal (i.e. attacking area not the attacking box). The spatial area of each zone was calculated using the maximum pitch dimensions outlined in the 2009/2010 FIFA ‘laws of the game’. Based on the assumption that referees spend most of the match time in the centre of the pitch, receiver proximity to each signal was determined by grouping the pitch zone data in the following factor levels: close (Dm & Am), near (Da & Aa) and far (Db & Ab).

The data set consisted of count, nominal and ordinal data. Whilst means and standard errors are presented in figures and descriptive statistics, nonparametric methods were used for all analyses. All correlational relationships between data were calculated using Spearman's rank correlation coefficient, rho. Analyses for both ‘potential outcomes for deceivers’ and ‘receiver proximity to signal’ used generalised linear mixed effect models (lmer) to determine significant effects of fixed factors on response variables; signal or reward counts (family =  Poisson) and dive or reward proportions (family =  Binomial), whereby ‘match’ was treated as a nested random factor. Whereas ‘negative frequency-dependent selection’ was analysed using generalised linear models (glm). The fixed factors (levels in brackets) tested were: pitch zone (Db, Da, Dm, Am, Aa and Ab), signaller team score (winning, drawing, losing), match time (1^st^,2^nd^, 3^rd^ and 4^th^ quarter), dive frequency (count data), league (A, B, C, D, E and F) and receiver proximity to signal (close, near, far). When determining the effects of pitch zone or proximity on dive use or reward frequency, dive and reward frequency were corrected for spatial area of zone (count/km^2^ area). Match time had no significant effect on dive use. Following significant effects in models, post-hoc pairwise comparisons using Tukey-Kramer HSD were calculated to determine significant differences between levels on the response variables. To determine significant differences between paired data on the rewarded proportion of dives versus tackles, for league and score, Paired-Wilcoxen signed rank tests were used with one-tailed tests reported for *P* values.

## Results

We predicted deception would be less common when the potential outcome for the deceiver is more costly relative to the potential benefit [15]. Thus, we expected dive frequency would decrease closer to the defensive goal, as goal-conceding opportunity (potential cost of deception) increases but goal-scoring opportunity (potential benefit of deception) decreases. In support of our prediction, dive frequency significantly decreased towards the defensive goal (dive frequency, rho = 0.282, *P*<0.001; relative to tackles, rho = 0.238, *P*<0.001). Further, relative to tackles, dives were significantly more common in the two closest zones to the attacking goal, than the zone closest to the defensive box (Fig. 1a, Db vs. Ab, Aa; *P* = 0.012; *P* = 0.041). Also, players dived twice as frequently when in the zone closest to the attacking goal than when in any defensive zone (Fig. 1b, Ab vs. Db, Da, Dm, *P*<0.001; *P*<0.001; *P* = 0.007). Similarly, in the second closest zone to the attacking goal, players dived more frequently than in any defensive zone (Fig. 1b, Aa vs. Db, Da, *P* = 0.006; *P* = 0.006). Dive frequency also increased towards the attacking goal despite referees rewarding proportionally fewer dives in those zones (rho = 0.228, *P*<0.011). Thus, deception in soccer players decreases towards the defensive goal where the potential outcome for the deceiver is more costly. Further, soccer players increase the use of deception towards the attacking goal where the potential outcome is most beneficial despite less chance of receiving the reward.

We investigated whether dive use by players would be affected by the signalling player's current score during a game. For instance, the potential benefit a player can gain from a dive is greatest when the score is even (goal-scoring opportunity to win match), less when losing (a goal-scoring opportunity to draw match) and lowest when winning (a goal-scoring opportunity to maintain win). As expected, significantly fewer dives occurred when players were winning or losing than when drawing (Fig. 1c, Drawing versus winning, losing, *P* = 0.007; *P* = 0.014). Dive frequency significantly decreased in the order of drawing, losing and winning (rho = 0.202, *n* = 60, *P* = 0.006). Soccer players therefore used deception when the potential outcome for the deceiver was most beneficial.

Receivers are expected to play an important role in limiting deception and thereby maintain honesty [15], [22]. Punishment of deceptive signallers by receivers can increase the potential cost of deception and thereby deter its use [5], [23]. We predicted that punishment administered by referees would be associated with a decrease in dives. However, of the 169 observed dives, none were punished by the referee. Of the 2633 tackles, 7 (0.3%) received a free-kick against the signalling player and 2 were punished with a yellow card. As such, no relationship between the punishment of deceivers and a decrease in the prevalence of deception was detected.

**Figure 2:**
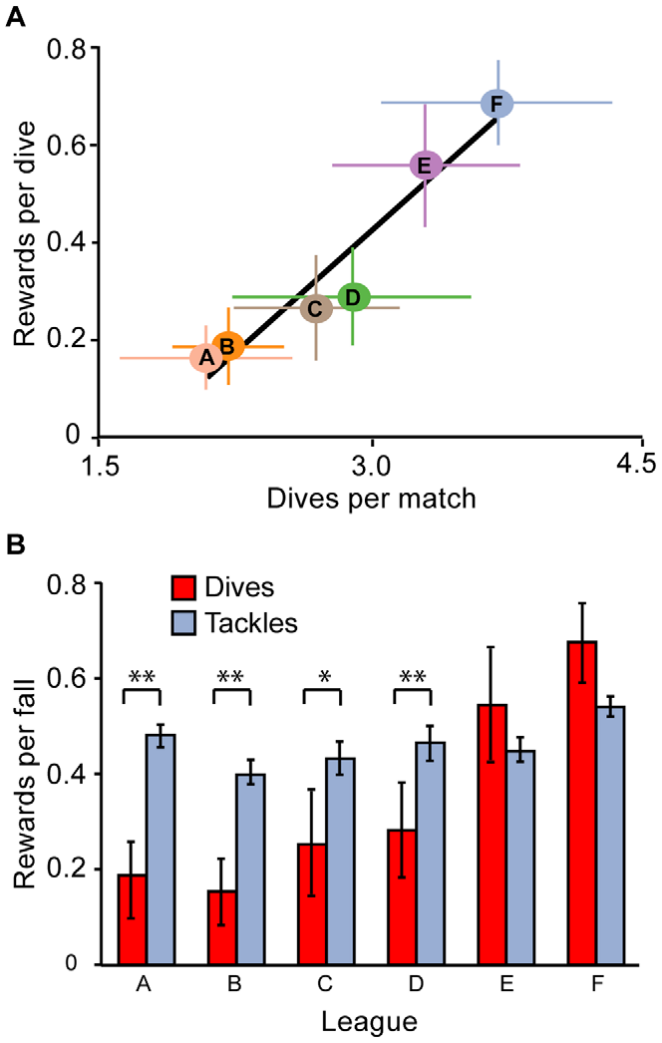
Detection of deception across leagues. (A) The mean proportion of total dives that were rewarded by the referee with a free-kick and the mean dive frequency of 10 matches in each league (*n* = 6). (B) Separated in to dives or tackles, the mean proportion that were rewarded by the referee across matches (*n* = 10) within each league. Leagues (A – F) are not identified due to ethical considerations. Standard error bars are presented. Black solid line indicates R^2^ however Spearman's rank correlation coefficient, rho_,_ was used to calculate significance. Asterisks denote significant differences based on Paired-Wilcoxen signed rank tests (*P*<0.05; **P<0.01; ***P<0.001).

Negative frequency-dependent selection is common in cases of animal mimicry [24], [25] whereby, deception is less successful when more frequent. We therefore predicted that dives by soccer players would be rewarded with a free-kick less often when more prevalent. Unexpectedly, dives were rewarded more often when they were more frequently used (by frequency, rho = 0.704, *P*<0.001; proportionally, rho = 0.372, *P* = 0.004). Also, the proportion of dives that were rewarded significantly varied across leagues (*F*_5,54_ = 5.367, *P*<0.001). The frequency of dives was positively associated with the proportion of dives rewarded by referees across leagues (Fig. 2a, rho = 1, *n* = 6, *P* = 0.003). League ‘F’ had significantly more dives rewarded than leagues A, B and C (Fig. 2a, F versus A, B, C, *P* = 0.006; *P* = 0.013; *P* = 0.045). We also determined whether different leagues rewarded proportionally fewer dives than tackles. Referees in leagues A, B, C and D rewarded significantly fewer dives than tackles (Fig. 2b, A, B, C, D, *P* = 0.002; *P* = 0.010; *P* = 0.044; *P* = 0.020). In contrast to our prediction, deception by soccer players was more successful when more common.

**Figure 3:**
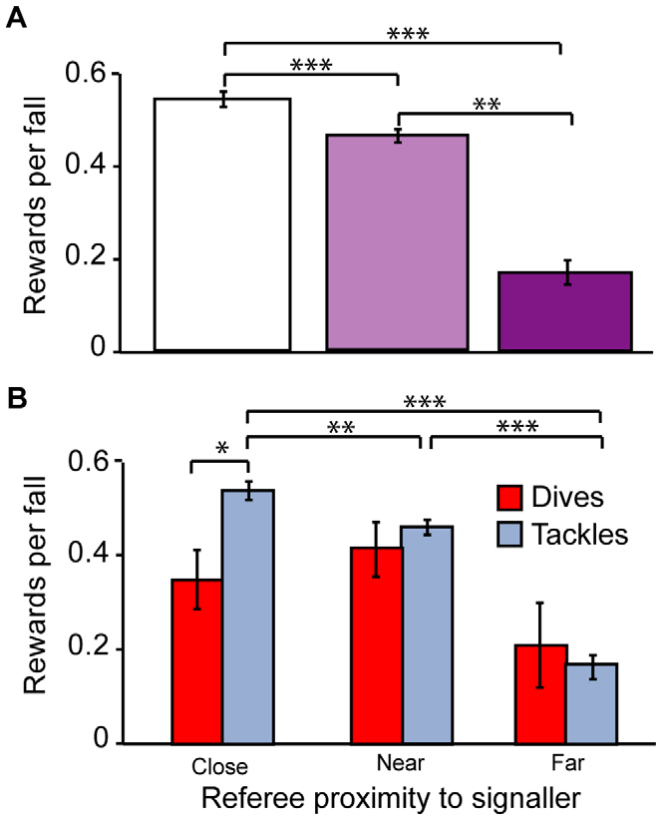
Detection of deception and referee proximity to signals. Receiver proximity is expressed as the referee being close, near or far to the fall signal. (A) The mean proportion of total falls that were rewarded by the referee per match across receiver proximity categories. (B) The mean proportions of total dives and tackles that were rewarded by the referee per match across receiver proximity categories. Standard error bars are presented (*n* = 60). Asterisks denote significant differences based on post-hoc Tukey-Kramer HSD (*P*<0.05; **P<0.01; ***P<0.001).

Spatial proximity of receivers to a signal is expected to improve the detection of signals [26]. Given that referees spend the majority of time in the centre of the pitch, we predicted that all fall signals by soccer players would be rewarded more often when they occurred closer to the referee. Referees rewarded more falls when closer to the signal (by frequency, rho = −0.669, *P*<0.001; proportionately, rho = −0.675, *P*<0.001). Falls that occurred close to the referee were three times more likely to be rewarded with a free-kick than those that occurred furthest away (i.e. goal boxes) (Fig. 3a, far vs. close, near, *P*<0.001; *P*<0.001; near vs. close, *P*<0.014). Tackles were also rewarded less often when the referee was further away from the signalling player (by frequency, rho = −0.696, *P*<0.001; proportionally, rho = −0.684, *P*<0.001). Proportionally, tackles that occurred furthest from the referee were rewarded the least, whereas those that were closer were rewarded most often (Fig. 3b, far tackles vs. close, near, *P*<0.001; *P*<0.001; near tackles vs. close, *P* = 0.006). Similarly, dives were rewarded less frequently when further from the referee (rho = −0.194, *P*<0.009). Referees were therefore less likely to respond to any fall when further away from the signaller. However, despite falls being rewarded more often when closer to the referee, deception did not significantly increase with closer proximity (rho = −0.071, *P* = 0.342).

Closer proximity to a signal is also expected to improve the ability of receivers to discriminate between deceptive and reliable signals [20], [21], [22]. We therefore predicted that the proportion of dives rewarded would be less that the proportion of tackles rewarded with a free-kick when in close proximity to the referee (i.e. better receiver discrimination ability). As predicted, when players were closest to the referee, a significantly lower proportion of dives were rewarded than tackles (Fig. 3b, close dives vs. tackles, *P*<0.005) but not when further away. Thus, closer proximity to signals was associated with improved signal detection [26] and discrimination ability by referees [20], [21], [22].

## Discussion

Our data suggests that humans are less likely to use deception when the potential cost for the deceiver is high relative to the potential benefit. Also, our results highlight that the ability to detect deception in humans may be associated with spatial proximity to the deceiver and cultural influences. Furthermore, cost-free deceit in soccer players provides empirical support to theories of animal signalling [15], [18], [19], [20], [21], [22].

The potential cost of deception may deter deceit by soccer players, whereas, the potential benefit of deception may act as an incentive. We found soccer players chose to deceive less frequently in defensive zones where the potential costly outcome was high relative to the potential benefit, and the inverse pattern also holds. However, these results do not allow us to distinguish whether it is the potential benefit or, the potential cost, that plays a stronger role in a player's decision to deceive. When considering the current score of the signalling player's team, deception was more common when the potential benefit was greatest (a draw score). However, this could be due in part to teams spending greater match time with a draw score. Overall, our results do suggest that humans are more likely to deceive when the potential outcome is highly beneficial, thereby outweighing the potential cost. Or conversely, when the potential outcome is very costly relative to the potential benefit, it may deter the use of deception. Interestingly, deceivers did not appear to take into account the likelihood of receiving a benefit, as dive frequency increased towards the attacking goal despite the referees rewarding proportionally fewer dives in those pitch zones. This pattern suggests that the potential benefit to deceivers may be a stronger incentive to deceive than the potential cost as a deterrent. Furthermore, the absence of punishment of deceivers by referees may also encourage the use of deception by soccer players.

The ability of referees to detect deception increased with closer proximity to the deceiver. Dives were rewarded less often relative to tackles when closest to the referee thereby supporting the prediction that receiver discrimination improves with closer proximity to the signaller [20], [21], [22]. Overall, more falls were rewarded when closer to the referee supporting the idea that signal detection improves with closer proximity to the signaller [21], [22], [26]. Another interpretation is that referees were reluctant to reward signals nearest to the goal, where the outcome for the signaller is highly costly, or beneficial. We also must acknowledge that linesmen occasionally contribute to the referee's decision-making and, the assumption that referees remain centrally on the pitch may not always hold true. Despite these caveats, our results do suggest that closer proximity to a deceiver can improve the detection of deception in soccer players.

Differences in the relative proportion of rewarded dives to tackles among leagues suggest there may be cultural differences in the ability of referees to discriminate deception or the ability of players to mimic tackles [9]. Further, the variation observed in discrimination ability by professional referees across leagues could be attributed to real-world cultural pressures because, under lab conditions non-referee observers can reliably distinguish between dives and tackle-induced falls [28]. Distinguishing among these alternative hypotheses opens up exciting avenues for further empirical analyses.

Professional soccer is a modern real-world human scenario, whereby soccer players intentionally deceive the referee by behaviourally mimicking a tackle-induced fall. Unlike tackles, dives are intentionally signalled and players can choose when to produce them [28]. The behavioural mimicry in this system therefore implies there is only the choice to deceive because opponents control tackle-induced falls (i.e. a reliable cue). Therefore, dive frequency reflects only changes in the decision to use deceptive signals. Note that when a referee rewards a dive, it is a clear case of undetected deception whereas a referee not rewarding a tackle-induced fall with a free-kick does not imply an undetected reliable cue because tackles can be either legal or illegal. As such, the strength of this study's empirical support for signalling theory is based on deceptive signalling and not the reliability of cues.

Our findings in a real-world human system provides empirical support of current signalling theory whereby, the potential cost for deceivers can maintain honesty under conflict of interest [15], [18], [19], [20], [21], [22]. In support of signalling theory [15], [18], the prevalence of cost-free deception in soccer players decreased as the potential outcome for deceivers was more costly relative to the potential benefit. In soccer, receiver proximity to signallers was associated with increased ability of receivers to discriminate and detect signals [21], [22], [26]. Deceptive signals by soccer players are cost-free and, the maintenance of honesty under conflict of interest was associated with how receivers respond and, the potential outcomes for deceivers. Furthermore, our results emphasise that receivers can play an important role in the maintenance of honesty under conflict of interest. Therefore our study of deception in humans supports theoretical models of animal signalling [15], [18], [20], [21], [22], [23], [26]. Furthermore, we hope this study has demonstrated the value of applying nonhuman research to human systems, to better understand human behaviour [29].
